# Impact of asymptomatic malaria infection on children’s growth in rural Malawi

**DOI:** 10.1038/s41598-025-13331-6

**Published:** 2025-08-01

**Authors:** Hany Sady, David Chaima, Lotta Hallamaa, Ulla Ashorn, Jomo Banda, Charles Mangani, John Kamwendo, Kenneth Maleta, Per Ashorn, Yue-Mei Fan

**Affiliations:** 1 Center for Child, Adolescent, and Maternal Health Research, Faculty of Medicine and Health Technology, Tampere University, Arvo Ylpön katu 34, Tampere, 33014 Finland; 2 Faculty of Medicine and Health Sciences, Hodeidah University, Hodeidah, Yemen; 3 School of Global and Public Health, Kamuzu University of Health Sciences, Blantyre, Malawi; 4 Department of Paediatrics, Tampere University Hospital, Tampere, Finland

**Keywords:** Asymptomatic malaria, *Plasmodium falciparum*, Malnutrition, Child growth, Malawi, Microbiology, Diseases, Health care, Medical research, Risk factors

## Abstract

Asymptomatic malaria infections are common in endemic regions, yet their impact on children’s growth remains inadequately understood. This study investigates the association between asymptomatic malaria and 6-18-month-old children’s growth indices in rural Malawi. Dried blood spots from 840 participants in Lungwena Child Nutrition Intervention 5 (LCNI-5) clinical trial were analysed at the baseline (*N* = 697) and every 3 months for a year. The associations between asymptomatic *Plasmodium falciparum* (determined by real-time PCR), and growth indices (length-for-age Z score (LAZ), weight-for-age Z score (WAZ), and weight-for-length Z score (WLZ)) were examined. Across all ages (6 to 18 months), malaria-positive children had lower mean WAZ (-1.03 vs. -0.87, 95% CI -0.17 − -0.04) and WLZ (-0.03 vs. -0.13, 95% CI -0.22 − -0.06) compared to those uninfected peers, whereas LAZ showed no significant association. However, no significant impact was observed at individual time points, except at 12 months of age. After adjusting for confounders, malaria infection remained associated with poorer children growth outcome. Asymptomatic malaria is linked to impaired growth outcomes among young children in rural Malawi. Targeted interventions aimed at managing asymptomatic malaria could mitigate growth faltering and improve child health in malaria-endemic settings.

**Supplementary Information:**

The online version contains supplementary material available at 10.1038/s41598-025-13331-6.

## Introduction

Malaria remains a formidable global health challenge in low-resource settings, particularly affecting young children and pregnant women^1^. In 2023, approximately 263 million malaria cases were reported worldwide, resulting in 597,000 deaths. Most of these cases (94%) and deaths (95%) occurred in the African region with 76% of the deaths occurring in young children^1^. While the clinical manifestations of malaria are well-documented, a significant proportion of infections in endemic regions are asymptomatic and often go unnoticed, posing a silent threat to children’s health and development^2,3^.

Malaria can be symptomatic or asymptomatic, with the latter involving *Plasmodium* parasites in the blood without symptoms and often going undetected^4^. Asymptomatic carriers in high-transmission areas have been found to become particularly infectious at the beginning of wet season^5^ and can rapidly develop symptoms, worsening the malaria burden and complicating control efforts^6^. These infections may persist^7^causing inflammation that diverts nutrients from growth to immune responses, adversely affecting children’s nutrition^8^raising the risk of undernutrition, anaemia, stunted growth, and cognitive deficits, often resulting in school absenteeism^9^. In parallel, early childhood is pivotal for growth, development, and long-term health outcomes, especially in endemic areas^10–12^. In rural Malawi, where malaria rates are high, and access to healthcare and food security are limited, many children under five suffer from stunting, underweight and wasting^13,14^. This underscores the urgent need for integrated interventions that address both malaria and undernutrition in these vulnerable populations.

Studies on the complex association between asymptomatic malaria and child growth in these settings have reported conflicting results that vary by both country and time, leaving the topic poorly understood^15,16^. For instance, study in Malawi found that *P. falciparum* infection indirectly triggers systemic inflammation by increased cytokines (TNF-α and IL-10) that inhibits critical growth hormone called insulin-like growth factor-1 (IGF-1) and finally associated with lower LAZ at 24 months of age^17^. Similarly, studies in Lao PDR (People’s Democratic Republic), Nigeria and Ghana found a significant association between asymptomatic malaria and stunting, suggesting impairing young children development^18–20^. While in 2025 a meta-analysis of 24 cross-sectional studies in Sub-Saharan Africa, revealed there was no significant association between chronic asymptomatic malaria and stunted school aged children^21^. The precise mechanisms through which asymptomatic malaria impedes children’s growth are intricate and multifaceted, encompassing both direct and indirect pathways. It has been hypothesised that *Plasmodium* parasites can impact the gastrointestinal tract, disrupting nutrient absorption and utilisation^22^and resulting in the malabsorption of essential nutrients, particularly iron and folate, which are vital for growth and development^23^. Furthermore, the chronic inflammation associated with asymptomatic malaria can interfere with the endocrine system, disrupting the production and regulation of growth-related hormones^24^. Indirectly, the burden of asymptomatic malaria strains already limited healthcare resources in rural Malawi, diverting attention and resources away from crucial interventions focused on child health and nutrition^25^.

Understanding the relationship between asymptomatic *P. falciparum* malaria and growth in young children is essential for public health efforts in malaria-endemic settings including Malawi. This study aims to investigate the effect of asymptomatic malaria infection on children’s growth in rural Malawian children aged 6–18 months. The present study hypothesises that asymptomatic *P. falciparum* malaria adversely influences anthropometric growth parameters weight-for-length Z-score (WLZ), weight-for-age Z-score (WAZ) and length-for-age Z-score (LAZ) over one-year follow-up.

## Methods

### Study design and population

This study was a secondary observational analysis of data and biological samples collected from the Lungwena Child Nutrition Intervention 5 (LCNI-5) clinical trial. The LCNI-5 conducted between 2008 and 2012 at and around Lungwena Health Centre and Malindi Hospital, in the lakeside district of Mangochi, South-Eastern Malawi and the protocol (NCT00524446) has been described in detail elsewhere^13^. In brief, of the 1385 infants assessed for study eligibility inclusion criteria, 840 healthy six-month-old infants were enrolled. They were randomised into four dietary intervention groups: standard treatment, fortified maize-soy flour, milk-based nutrient supplement, or soy-based nutrient supplement and were followed up for 12 months. Child growth was monitored at 3-month intervals and dry blood spots (DBS) were collected during the same visits. The study protocol was registered in the clinical trial registry of the National Library of Medicine, Bethesda, MD, USA (http://www.clinicaltrials.gov, trial identification NCT00524446).

For inclusion criteria in the LCNI-5 trial, healthy infants aged between 5.5 and 6.5 months, permanent residents of the catchment area and available during the study period. Eligible infants were those with no severe illness or history of anaphylaxis that would warrant hospital referral. Infants with WLZ who had less than 80% of the WHO reference median (typically corresponds to WLZ< −2) or who showed signs of oedema were excluded.

The trial was performed according to Good Clinical Practice guidelines and the ethical standards of the Helsinki Declaration. The protocol was approved by the College of Medicine Research and Ethics Committee, Malawi, and the Ethical Committee of Pirkanmaa Hospital District, Finland. Only participants whose guardians signed informed consent forms were enrolled in the study.

### Sample collection, DNA extraction and real-time PCR

Participants were seen at the health centre at enrolment when they were 6 months old and every 3 months for a year, undergoing a clinical check-up and venous blood sampling. From the blood samples taken at the clinic, 100 µl (2 spots, each 50 µl) was applied to Whatman FTA filter paper (Whatman plc, Maidstone, UK), air-dried, and placed in individually sealed plastic bags with a desiccant. The sample bags were then stored in dry condition at room temperature for about 5 years before the analyses. Genomic DNA was extracted from the DBS samples using a previously published method^26^. Real-time PCR (rt-PCR) was used to detect the lactate dehydrogenase gene (LDH) of *P. falciparum* as described elsewhere^27^. All reactions were run in duplicate on a StepOne plus PCR instrument (Applied Biosciences, USA). Each reaction plate included four serial dilutions of *P. falciparum* 3D7 genomic DNA as positive controls and a no template control. Samples were considered positive if both samples had a cycle threshold (CT) value of < 45. Reactions with only one sample having a CT value were repeated if the sample was available.

*Plasmodium* infections were considered asymptomatic when a participant’s PCR result was positive. The caregiver self-reported their child’s health status by using a weekly morbidity calendar form for LCNI-5 (see additional file 1). Morbidity form recorded each day by the guardians in a picture calendar, indicating if the child was healthy or had symptoms for fever, coughing, and diarrhoea. The information collected from the guardians during the biweekly food delivery. Additional information on the participants were collected by the research assistants, carrying out a limited health assessment of the participant during their biweekly home visits.

### Anthropometric measurements

Three research assistants carried out anthropometric measurements during the enrolment visit. Unclothed infants were weighed using an electronic infant weighing scale (SECA 735; Chasmors Ltd. London, England) and weights were recorded to the nearest 10 g. The length was measured to the nearest 1 mm using a high-quality length board (Kiddimetre; Raven Equipment Ltd, Essex, England). We calculated age- and sex-standardized anthropometric indices (LAZ, WAZ and WLZ) using the STATA macro developed by WHO using the WHO 2006 multicentre growth standards^28^.

### Collection of baseline background information and haemoglobin measurements

The participants’ socioeconomic background data were collected through interviews with their caregivers during enrolment. At enrolment, the participants’ blood haemoglobin concentration was also measured from a venous sample using a HemoCue device (HemoCue AB, Angelholm, Sweden).

#### Statistical analysis

The baseline characteristics of participants were summarised as mean with standard deviation (SD) for continuous variables and as percentages for categorical variables. Differences between included and excluded participants were assessed using Student’s t-test for continuous variables and Fisher’s exact test for categorical variables.

The analysis included all participants with data on growth and DBS samples collected during the visits and have a PCR results. Malaria status was defined as negative or positive with the PCR results at each time point when DBS sample was available. A linear mixed-effects model was used as a primary analysis to estimate child growth based on malaria status. All participants with at least one growth measurement and malaria sample during follow-up were included in the analyses. The mixed-effects model adjusts for the effect of missing values by using the correlation between repeated measurements and can thus be used to estimate parameters without imputing the missing data, even if only one measurement exists^29^. The model included random intercepts by participant and specified a first order autoregressive residual structure to account for waning within-child correlations in anthropometric measurements over time (weaker correlation between distant vs. adjacent measurements). This structure was chosen based on biological plausibility and evaluation of the covariance pattern. Discrete analyses were also carried out as a secondary analysis for each time point (6, 9, 12, 15, and 18 months). Ordinary least squares regression was used to examine the mean differences in WLZ, WAZ and LAZ separately between children with positive and negative malaria test results. Results were presented as a mean (SD) and 95% confidence intervals (CI). A P-value of less than 0.05 was considered statistically significant. The adjusted analysis controlled for maternal education, child sex, the season of visit, enrolment site, intervention group, nutritional status, bed net use, WLZ and haemoglobin concentration at enrolment.

For secondary analyses of individual time points, we applied Holm’s method to control type I error risk, adjusting comparisons for multiplicity. Specifically, analyses of the three growth Z-scores (WAZ, WLZ, and LAZ) were adjusted for five tests (one per time point). While primary analyses compared differences between malaria positive or negative children across all time points and did not require multiplicity adjustment.

To address temporality, we conducted post-hoc analyses testing associations between malaria status at 6, 9, 12, and 15 months and anthropometric outcomes three months later. Models were adjusted for the same covariates as concurrent analyses, with additional adjustment for the baseline value of the respective growth indicator to account for prior nutritional status. We also conducted analyses using ordinary least squares regression to assess whether any malaria status between 6 and 15 months predicts growth at 18 months. All statistical analyses were conducted using Stata version 17.0 (Stata Corp, College Station, USA).

## Results

### Characteristics of participants at baseline

Out of the 840 enrolled infants, DBS samples were collected from 697 participants at the study baseline (6 months of age), and these participants were followed up until 18 months of age. The number of DBS samples collected during each visit at 6, 9, 12, 15, and 18 months was 697, 553, 515, 436, and 545 respectively. At baseline, the total group of 840 infants had an equal distribution of boys and girls. They had an average weight of 6.9 kg and an average length of 62.9 cm. Their mean LAZ, WAZ and WLZ were − 1.69, −0.80 and 0.46 respectively. On average, their haemoglobin concentration was 95 g/L. The mothers of these infants had an average of 3.5 years of education, and 72.5% of the families used bed nets. The baseline characteristics of included and excluded participants were similar, except that the excluded participants were on average slightly older and taller (Table 1). Approximately 17% of the study cohort participants were excluded from the analysis, due to failure to attend scheduled clinic visits or refusal to provide the required samples.

**Table 1:** Baseline characteristics of participants.

Participant characteristics	included	excluded	*P* value
Number of participants	697	143	
Infant male sex (%)	49.4%	46.9%	0.65
Age, months	6.0 (0.2)	6.1 (0.2)	< 0.001
Weight, kg	7.0 (0.9)	7.1 (1.0)	0.13
Length, cm	63.0 (2.2)	63.6 (2.4)	0.004
Weight-for-age z score	−0.8 (1.1)	−0.7 (1.1)	0.31
Length-for-age z score	−1.7 (1.0)	−1.5 (1.0)	0.04
Weight-for-length z score	0.5 (1.0)	0.4 (1.1)	0.67
Socio-economic score	−0.01 (1.0)	0.06 (1.2)	0.41
Maternal education, years	3.4 (3.2)	3.8 (3.5)	0.23
Bed net use (%)	72.5%	76.7%	0.34
Haemoglobin (g/L)	94.6 (17.0)	95.3 (15.7)	0.64

Data are presented as mean (SD) or percentage (%).

P-value obtained from Student’s t-test or Fisher’s exact test.

### Association between peripheral blood malaria status and infant’s anthropometric indices (LAZ, WAZ and WLZ)

The overall prevalence of asymptomatic malaria across all study visits was 13.9%, with the highest prevalence at 18 months of age (22.4%) compared to 14.1% at enrolment^30^.

When data from all study visits were examined, mean WAZ and WLZ were found significantly lower in children who tested positive for malaria compared to those who tested negative (Table 2) while no significant association was observed for LAZ. The mean (SD) WAZ was − 1.03 (1.09) for malaria-positive participants and − 0.87 (1.06) for malaria-negative participants, with a mean difference of −0.07 (95% CI −0.13 to −0.02). Similarly, the mean WLZ was − 0.03 (1.07) for malaria-positive participants, compared to 0.13 (1.04) for malaria-negative ones, with a mean difference of −0.09 (95% CI −0.17 to −0.01). The mean LAZ was not significantly different (−1.85 vs. −1.74, 95% CI 0.10 − −0.01) in participants who were positive for malaria infection compared to their negative peers when data from all study visits were examined. However, there were no significant differences in WAZ, WLZ and LAZ between malaria-positive and negative groups at 6, 9, 15 and 18 months of age (Tables 3, 4 and 5).

**Table 2:** The association between malaria parasitemia and attained anthropometric indicators across all study visits.

Child age (across all study visits)	Unadjusted Mean (SD) attained WAZ, WLZ & LAZ by the participants’ malaria test result				
	Children with negative malaria test result	Children with positive malaria test result	Difference between groups (95% CI)	^a^*P*-value	^b^*P*-value
n	2358	385			
WAZ	−0.87 (1.06)	−1.03 (1.09)	−0.07 (−0.13, −0.02)	**0.009**	**0.014**
WLZ	0.13 (1.04)	−0.03 (1.07)	−0.09 (−0.17, −0.01)	**0.021**	**0.019**
LAZ	−1.74 (1.02)	−1.85 (1.06)	−0.05 (−0.10, 0.01)	0.094	0.092

WAZ, weight-for-age Z score; WLZ, weight-for-length Z score; LAZ, length-for-age Z score.

^a^P-value obtained from mixed-effects model for all time points.

^b^P-value obtained mixed-effects model for all time points. Adjusted for maternal education, child sex, the season of visit, enrolment site, intervention group, bed net use, WLZ and haemoglobin concentration at enrolment.

**Table 3:** The association between malaria parasitemia and attained weight-for-age Z-score (WAZ) at 6, 9, 12, 15 and 18 months of age.

Child age (*n*, negative/positive)	Unadjusted Mean (SD) attained WAZ by the participants’ malaria test result					
	Children with negative malaria test result	Children with positive malaria test result	Difference between groups^a^ (95% CI)	Difference between groups^b^ (95% CI)	^a^*P*-value	^b^*P*-value
6 months (596/98)	−0.78 (1.12)	−0.95 (1.06)	−0.17 (−0.40, 0.07)	−0.004 (−0.16, 0.16)	0.17	0.96
9 months (505/48)	−0.82 (1.07)	−0.76 (1.14)	0.06 (−0.26, 0.38)	−0.12 (−0.38, 0.13)	0.73	0.35
12 months (457/58)	−0.84 (1.05)	−1.29 (1.28)	−0.45 (−0.74, −0.15)	−0.28 (−0.52, −0.04)	**0.003**	**0.02**
15 months (378/58)	−0.94 (1.02)	−1.09 (1.11)	−0.15 (−0.43, 0.14)	−0.03 (−0.29, 0.23)	0.31	0.82
18 months (422/123)	−1.00 (1.02)	−1.05 (0.98)	−0.05 (−0.25, 0.15)	−0.01 (−0.21, 0.19)	0.64	0.94

^a^P-value obtained from ordinary least squares for individual time points.

^b^P-value obtained from ordinary least squares for individual time points. Adjusted for maternal education, child sex, the season of visit, enrolment site, intervention group, bed net use, WLZ and haemoglobin concentration at enrolment.

**Table 4:** The association between malaria parasitemia and attained weight-for-length Z-score (WLZ) at 6, 9, 12, 15 and 18 months of age.

Child age (*n*, negative/positive)	Unadjusted Mean (SD) attained WLZ by the participants’ malaria test result					
	Children with the negative malaria test result	Children with the positive malaria test result	Difference between groups^a^ (95% CI)	Difference between groups^b^ (95% CI)	^a^*P*-value	^b^*P*-value
6 months (596/98)	0.48 (1.04)	0.30 (1.07)	−0.18 (−0.40, 0.04)	−0.05 (−0.21, 0.10)	0.11	0.49*
9 months (505/48)	0.17 (1.02)	0.32 (1.10)	0.14 (−0.16, 0.45)	−0.09 (−0.29, 0.10)	0.36	0.35
12 months (457/58)	0.04 (1.00)	−0.35 (1.05)	−0.39 (−0.66, −0.11)	−0.25 (−0.45, −0.05)	**0.006**	**0.014**
15 months (378/58)	−0.11 (0.94)	−0.31 (1.01)	−0.19 (−0.46, 0.07)	−0.08 (−0.30, 0.13)	0.15	0.45
18 months (422/123)	−0.10 (1.04)	−0.16 (0.99)	−0.06 (−0.27, 0.14)	−0.01 (−0.21, 0.18)	0.56	0.90

^a^P-value obtained from ordinary least squares for individual time points.

^b^P-value obtained from ordinary least squares for individual time points. Adjusted for maternal education, child sex, the season of visit, enrolment site, intervention group, bed net use, WLZ (*not at 6 months, instead WAZ) and haemoglobin concentration at enrolment.

**Table 5:** The association between malaria parasitemia and attained length-for-age Z-score (LAZ) at 6, 9, 12, 15 and 18 months of age.

Child Aae (*n*, negative/positive)	Unadjusted Mean (SD) attained LAZ by the participants’ malaria test result					
	Children with the negative malaria test result	Children with the positive malaria test result	Difference between groups^a^ (95% CI)	Difference between groups^b^ (95% CI)	^a^*P*-value	^b^*P*-value
6 months (596/98)	−1.69 (1.02)	−1.73 (0.99)	−0.05 (−0.26, 0.17)	−0.01 (−0.24, 0.22)	0.68	0.93
9 months (505/48)	−1.62 (1.04)	−1.72 (1.01)	−0.10 (−0.41, 0.20)	−0.10 (−0.42, 0.22)	0.51	0.55
12 months (457/58)	−1.71 (1.00)	−1.98 (1.31)	−0.27 (−0.56, 0.01)	−0.14 (−0.43, 0.15)	0.06	0.34
15 months (378/58)	−1.80 (1.04)	−1.82 (1.05)	−0.02 (−0.30, 0.27)	0.05 (−0.26, 0.36)	0.91	0.75
18 months (422/123)	−1.96 (0.98)	−1.95 (1.02)	0.01 (−0.19, 0.21)	0.02 (−0.19, 0.24)	0.90	0.85

^a^P-value obtained from ordinary least squares for individual time points.

^b^P-value obtained from ordinary least squares for individual time points. Adjusted for maternal education, child sex, the season of visit, enrolment site, intervention group, bed net use, WLZ and haemoglobin concentration at enrolment.

At 12 months, mean WAZ and WLZ values were notably lower in malaria-positive participants than in negative ones, with a mean difference of −0.45 (95% CI −0.74 − −0.15) for WAZ and − 0.39 (95% CI −0.66 − −0.11) for WLZ (Tables 3 and 4). There were no significant differences in LAZ between the two groups at individual time points.

Adjustments for covariates including maternal education, child sex, the season of visit, enrolment site, intervention group, bed net use, nutritional status (WLZ) and haemoglobin concentration at enrolment did not markedly change the results. The observed differences remained consistent after adjustment for these factors. Furthermore, all significant associations observed in unadjusted analyses remained statistical significance following Holms’s adjustment for multiple testing.

In post-hoc analyses assessing delayed effects of malaria on growth, parasitemia at 12 months was associated with lower WAZ (−1.37 vs. −0.96; 95% CI: −0.72 − −0.10) and WLZ (−0.51 vs. −0.15; 95% CI: −0.66 − −0.07) at 15 months in unadjusted models. However, after adjustment for the above covariates and WAZ/WLZ at 12 months, all associations became non-significant. No significant associations were observed for parasitemia at 6, 9, or 12 months with subsequent WLZ, WAZ, or LAZ in either unadjusted or adjusted models (Supplementary Tables S1-S3). We used ordinary least squares regression to assess whether any malaria status between 6 and 15 months predicts growth at 18 months. No significant associations were found with or without adjustment for covariates (Supplementary Table S4).

## Discussion

The present study aimed to test the hypothesis that asymptomatic malaria infection is associated with reduced growth in children aged 6 to 18 months. Our findings demonstrated a significant association between peripheral blood malaria parasitemia detected by PCR assay, and children’s anthropometric measurements (WAZ and WLZ, but not LAZ). These results support the hypothesis proposed in this study, as infants experiencing asymptomatic malaria exhibited poorer growth outcomes between 6 and 18 months of age, with the effect being particularly pronounced at 12 months of age.

One of the present study limitations is the prolonged storage duration of DBS cards at room temperature prior to the PCR assays, which might have compromised the integrity of the genomic DNA extracted from the samples, potentially leading to underestimating *P. falciparum* prevalence. This could affect the accuracy and reliability of the associations between *P. falciparum* infection and growth outcomes. Additionally, confounders such as co-infection and other infections could introduce bias due to the influence of unmeasured factors. To account for variations in the number of observations per participant, we employed a mixed-effects model that incorporates participants’ intercepts as random effects. This model adjusts for missing values by using the correlation between repeated measurements, enabling accurate parameter estimation, even when only a single measurement exists for some participants^29^. Moreover, concurrent measurement of anthropometry and malaria limits causal inference due to temporality. However, this approach prioritized assessing acute effects (nutrient diversion), with baseline WLZ adjustments reducing reverse causality concerns. Post-hoc analyses showed no significant associations after adjustment for confounders, suggesting acute impacts may be obscured by socioeconomic or environmental factors.

On the other hand, approximately 17% of participants excluded from the study analysis at baseline due to either failure to attend scheduled clinic visit or refusal to provide the required samples. However, the excluded participants were mostly similar to those included in the analysis, aside from being slightly older and taller. Our study cohort was drawn from children enrolled in a nutritional-intervention clinical trial with frequent follow-up visits, which enhances internal validity but may limit generalizability to broader populations in resource-limited settings lacking similar trial infrastructures. Relatively small sample size at individual timepoints necessitated pooling data across visits, potentially obscuring time-specific effects. Nevertheless, we believe these findings provide valuable insights into understanding the relationship between asymptomatic malaria and early childhood growth, particularly in settings with similar health interventions or systematic healthcare frameworks.

Our findings are in agreement with a few previous studies that have documented the prevalence of asymptomatic malaria among children under five years of age and its association with poor growth^17,18,20,31–33^. For instance, a recent study involving Malawian infants found an association between asymptomatic *P. falciparum* infection before six months of age and a lower WAZ score during follow-up^31^. Similar findings emerged in rural Malawi, involving children aged 6–18 months, residing in a setting with a high prevalence of malaria and undernutrition, reported no association between malaria and changes in LAZ^34^. Additionally, studies from Tanzania and Mali identified a significant correlation between asymptomatic malaria parasitemia and underweight among young children compared to their uninfected counterparts^32,33^. Furthermore, research conducted in Ghana and Lao People’s Democratic Republic found that asymptomatic malaria was associated with stunted growth in school-aged children^18,20^.

Our results suggest that asymptomatic malaria may hinder growth during late infancy (6 to 18 months), leading to stunting, and underweight children which in turn may increase vulnerability to malaria. Previous studies support a causal link, showing growth impairment following malaria exposure. Biologically, since asymptomatic infections often evade immune detection and remain untreated with prolonged exposure in endemic regions^35^malaria may induce growth restriction through chronic inflammation, metabolic disruptions, and nutrient absorption issues^8,24^. However, confounding factors such as socioeconomic status^36^ and co-infection^37^could challenge direct causality claims.

Earlier studies have reported inconsistent results regarding the association between asymptomatic malaria infection and growth outcomes^34,38–41^. For example, a study in rural Cameroon found no correlation between asymptomatic malaria parasitaemia and underweight status among children under fourteen years of age^38^. Similarly, research in rural Kenya didn’t establish a clear association between wasting or stunting and asymptomatic malaria in children aged 2 to 36 months^39^. Other studies from Ghana showed that asymptomatic malaria had no significant impact on the anthropometric measures of children under five years^40,41^. Likewise, research in rural Malawi, where an indirect association was identified between malaria infections and diminished LAZ at 24 months among asymptomatic children aged 18–24 months^17^.

Our study found that asymptomatic malaria significantly impacted WAZ and WLZ at 12 months, indicating a critical period where growth is particularly pronounced. This finding aligns with existing research that suggests fetuses in malaria-endemic areas may acquire temporary adaptive immunity from prenatal exposure with the protective antibodies then gradually drop after 6 to 12 months of infant birth^42,43^. As immunity wanes, malaria prevalence increases and impacts growth. Notably, the impact of asymptomatic malaria on LAZ was not pronounced at any time points compared to WAZ and WLZ. This may be because WAZ and WLZ are more sensitive to short-term changes serving as a quicker predictor during illness or malnutrition^44^. In contrast, LAZ is slower to react and reflects long-term growth patterns and chronic factors like undernutrition or repeated infections, particularly in the first two years of life^45,46^.

The inconsistency between previous studies and our current findings regarding the correlation between asymptomatic malaria and children’s growth may be influenced by several factors. These factors include variations in study sample sizes, the methods used for detecting asymptomatic malaria, seasonality, study population characteristics, differences in socioeconomic level, and nutritional status. These varied results highlight the need for further studies to elucidate the complex relationship between asymptomatic malaria infection and growth outcomes such as LAZ. Future studies could explore the connection between asymptomatic infections and biomarkers of systemic inflammation, such as C-reactive protein and alpha-1-acid glycoprotein, as well as growth-related biomarkers like insulin growth factor. Additionally, it would be valuable to investigate other contributing factors to growth retardation in young children living in malaria-endemic areas, including exposure to enteropathogens.

In conclusion, our findings support the study hypothesis that asymptomatic malaria infection negatively influences growth in relation to weight in infants aged 6 to 18 months, with a heightened effect observed at 12 months. These results underscore the importance of timing interventions during critical developmental periods to address the detrimental effects of asymptomatic malaria on child health and growth.

## Supplementary Information

Below is the link to the electronic supplementary material.

Supplementary Material 1

Supplementary Material 2

## Data Availability

Data described in the manuscript, codebook, and analytic code will be made available upon request pending approval by the corresponding author, Hany Sady.

## Abbreviations

DBS: dry blood spot
Plasmodium falciparum: P. falciparum
rt-PCR: real-time polymerase chain reaction
LCNI-5: Lungwena Child Nutrition Intervention 5
LDH: lactate dehydrogenase gene
LAZ: length-for-age Z score
WAZ: weight-for-age Z score
WLZ: weight-for-length Z score
SD: standard deviation
WHO: World Health Organization
CI: confidence interval

## Declarations

### Supplementary Information

Additional file 1: weekly morbidity calendar form for LCNI-5 study.
