# Supplementary material for: Impact of asymptomatic malaria infection on children’s growth in rural Malawi
Source: Sci Rep. 2025 Aug 1;15:28094. doi: 10.1038/s41598-025-13331-6 (PMC12317011; doi:10.1038/s41598-025-13331-6)
Supplement: Supplementary file 1 — Supplementary Material 1 [file 41598_2025_13331_MOESM1_ESM.docx]

**Supplementary Table 1.** **The association between malaria parasitemia at 6, 9, 12, and 15 months of age and weight-for-age Z-score (WAZ) in the subsequent 3 months**

| **Child Age (n, negative/positive)** | **Unadjusted Mean (SD) attained WAZ by the participants’ malaria test result** | | | | |
| --- | --- | --- | --- | --- | --- |
|  | **Children with negative malaria test result** | **Children with positive malaria test result** | **Difference between groups (95% CI)** | **^a^P-value** | **^b^P-value** |
| 9 months (486/78) | -0.82 (1.08) | -0.82 (1.02) | -0.001 (-0.26, 0.26) | 0.99 | 0.99 |
| 12 months (428/42) | -0.88 (1.07) | -0.85 (1.11) | 0.03 (-0.32, 0.37) | 0.88 | 0.90 |
| 15 months (396/47) | -0.96 (1.02) | -1.37 (1.13) | -0.41 (-0.72, -0.10) | **0.01** | 0.63 |
| 18 months (381/53) | -0.96 (1.00) | -1.08 (0.89) | -0.13 (-0.41, 0.16) | 0.38 | 0.87 |

^a^P-value obtained from ordinary least squares for individual time points.

^b^P-value obtained from ordinary least squares for individual time points. Adjusted for maternal education, child sex, the season of visit, enrolment site, intervention group, bed net use, WLZ and haemoglobin concentration at enrolment and WAZ at the same visit as malaria status.

**Supplementary Table 2.** **The association between malaria parasitemia at 6, 9, 12, and 15 months of age and weight-for-length Z-score (WLZ) in the subsequent 3 months**

| **Child Age (n, negative/positive)** | **Unadjusted Mean (SD) attained WLZ by the participants’ malaria test result** | | | | |
| --- | --- | --- | --- | --- | --- |
|  | **Children with the negative malaria test result** | **Children with the positive malaria test result** | **Difference between groups (95% CI)** | **^a^P-value** | **^b^P-value** |
| 9 months (486/77) | 0.17 (1.03) | 0.24 (0.84) | 0.06 (-0.18, 0.31) | 0.60 | 0.24 |
| 12 months (428/42) | 0.02 (1.00) | 0.16 (0.98) | 0.14 (-0.18, 0.46) | 0.39 | 0.94 |
| 15 months (396/47) | -0.15 (0.98) | -0.51 (0.87) | -0.36 (-0.66, -0.07) | **0.015** | 0.25 |
| 18 months (381/53) | -0.05 (0.99) | -0.19 (0.92) | -0.14 (-0.42, 0.14) | 0.56 | 0.80 |

^a^P-value obtained from ordinary least squares for individual time points.

^b^P-value obtained from ordinary least squares for individual time points. Adjusted for maternal education, child sex, the season of visit, enrolment site, intervention group, bed net use, WAZ and haemoglobin concentration at enrolment and WLZ at the same visit as malaria status.

**Supplementary Table 3.** **The association between malaria parasitemia at 6, 9, 12, and 15 months of age and length-for-age Z-score (LAZ) in the subsequent 3 months**

| **Child Age (n, negative/positive)** | **Unadjusted Mean (SD) attained LAZ by the participants’ malaria test result** | | | | |
| --- | --- | --- | --- | --- | --- |
|  | **Children with the negative malaria test result** | **Children with the positive malaria test result** | **Difference between groups (95% CI)** | **^a^P-value** | **^b^P-value** |
| 9 months (486/77) | -1.62 (1.03) | -1.66 (1.07) | -0.04 (-0.29, 0.21) | 0.78 | 0.64 |
| 12 months (428/42) | -1.74 (1.06) | -1.89 (1.14) | -0.15 (-0.49, 0.19) | 0.38 | 0.88 |
| 15 months (396/47) | -1.80 (1.00) | -2.04 (1.34) | -0.24 (-0.56, 0.07) | 0.13 | 0.65 |
| 18 months (381/53) | -1.94 (0.98) | -1.98 (1.00) | -0.04 (-0.32, 0.25) | 0.80 | 0.75 |

^a^P-value obtained from ordinary least squares for individual time points.

^b^P-value obtained from ordinary least squares for individual time points. Adjusted for maternal education, child sex, the season of visit, enrolment site, intervention group, bed net use, WLZ and haemoglobin concentration at enrolment and LAZ at the same visit as malaria status.

**Supplementary Table 4. The association between malaria parasitemia at 6 to 15 months of age and growth indicators at 18 months**

| **Growth indicators (n, negative/positive)** | **Unadjusted Mean (SD) attained LAZ by the participants’ malaria test result** | | | | |
| --- | --- | --- | --- | --- | --- |
|  | **Children with the negative malaria test result** | **Children with the positive malaria test result** | **Difference between groups (95% CI)** | **^a^P-value** | **^b^P-value** |
| WAZ (486/77) | -1.03 (1.06) | -1.04 (0.95) | -0.01 (-0.17, 0.15) | 0.92 | 0.42 |
| WLZ (428/42) | -0.16 (1.06) | -0.11 (0.98) | 0.05 (-0.11, 0.21) | 0.54 | 0.16 |
| LAZ (495/245) | -1.92 (1.00) | -2.01 (1.34) | -0.08 (-0.24, 0.07) | 0.29 | 0.31 |

^a^P-value obtained from ordinary least squares for individual time points.

^b^P-value obtained from ordinary least squares for individual time points. Adjusted for maternal education, child sex, the season of visit, enrolment site, intervention group, bed net use, WLZ and haemoglobin concentration at enrolment and growth indicator at enrolment.
